# Plastic Deformation of Pressured Metallic Glass

**DOI:** 10.3390/ma10121361

**Published:** 2017-11-27

**Authors:** Yun Cheng, Chuanxiao Peng, Zhenting Zhang, Pengfei Wang, Shengzhong Yuan, Li Wang

**Affiliations:** School of Mechanical, Electrical & Information Engineering, Shandong University (Weihai), 180 Wenhua Xi Road, Weihai 264209, China; chy81@wh.sdu.edu.cn (Y.C.); believechx@163.com (C.P.); zhangzhentingzzt@163.com (Z.Z.); eloo@sohu.com (P.W.); ysz@sdu.edu.cn (S.Y.)

**Keywords:** metallic liquid, high pressure, plastic deformation, dynamical heterogeneity

## Abstract

Although pressured metallic glass (MG) has been reported in the literature; there are few studies focusing on pressure effects on the structure; dynamics and its plastic deformation. In this paper; we report on and characterize; via molecular dynamics simulation, the structure and dynamics heterogeneity of pressured MGs, and explore a causal link between local structures and plastic deformation mechanism of pressured glass. The results exhibit that the dynamical heterogeneity of metallic liquid is more pronounced at high pressure, while the MGs were less fragile after the release of external pressure, reflected by the non-Gaussian parameter (NGP). High pressure glass shows better plastic deformation; and the local strain zone distributed more uniformly than of in normal glass. Further research indicates that although the number of icosahedrons in pressured glass was much larger than that in normal glass, while the interpenetrating connections of icosahedra (ICOI) exhibited spatial correlations were rather poor; In addition, the number of ‘fast’ atoms indexed by the atoms’ moving distance is larger than that in normal glass; leading to the sharp decreasing in number of icosahedrons during deformation. An uniform distribution of ‘fast’ atoms also contributed to better plastic deformation ability in the pressured glass. These findings may suggest a link between the deformation and destruction of icosahedra with short-range order.

## 1. Introduction

Since their discovery several decades ago, metallic glasses (MGs) have attracted extensive attention in academia due to their excellent mechanical properties [1,2,3,4]. It is well known that the mechanical properties of the MGs are dependent on their internal structure, which may be influenced by changing the processing history, such as using different cooling rate for MG preparation [5,6,7]. Recently, some researchers have found that MGs obtained under high hydrostatic pressure possessed a high-density, are well-ordered, and possess a high-energy metallic glass phase which contradicts the common understanding of MGs [8,9,10,11,12,13,14,15]. While the relationship between the pressure and the internal structure is not well understood, and the mechanisms of shear band formation and propagation in pressured MGs are rarely discussed. The major challenge is to clarify structural heterogeneity in pressured MGs, and to establish a causal link between key local structures and macroscopic mechanical properties. High pressure is in favor of the formation of glass due to its inhibition of atom movement. Based on conventional free volume theory, the number of full icosahedra (FI) decreases in the less-relaxed state, which is usually realized by rapid cooling [7,8]. It is pointed that the number of full icosahedra increases with increasing pressure and the formed MG becomes denser, as expected [13]. More importantly, the atomic structure is remarkably mediated in virtue of pressure, which is identified by the Voronoi tessellation analysis. Since the pressure could also slow down the dynamics of the metallic liquid, leading to glass, the metallic liquid must be stronger, with higher glass formation ability (GFA). Roland et al. also find that the isobaric fragility always decreases with increasing pressure, although the pressure range considered is limited [14,15]. Further more, the deformability of metallic glasses is also improved after pressure-promoted rejuvenation processes by increasing the characteristic short- and medium range order, even though it leads to a higher-energy glassy state [16]. While recent computer simulations show that pressure makes a model liquid more fragile, which is also related to the dynamics heterogeneity [17]. The enhancement of the locally preferred structure with higher five-fold symmetry with increasing pressure may indicate a more complicated free energy profile for the system, and thus, a more intricate potential energy landscape with numerous well-separated ‘megabasins’. As a consequence, the more fragile liquid under high pressures is more dynamically heterogeneous [18].

Since the icosahedron is the typical cluster in the liquid and glass, the number and connectivity type may be connected with the increasing fragility under pressures. Strong evidence has been found to suggest that the icosahedral clusters often interpenetrate one another in Cu-Zr liquids, so that five atoms coincide on two icosahedral shells. These interpenetrating connections of icosahedra (ICOI) exhibit strong spatial correlations [19,20] and produce stable string-like networks of icosahedra that are uniquely responsible for dynamic slowing during the formation of the Cu-Zr MGs [3,20,21]. They gradually grow and form complex network to hinder the atomic long-distance moving until the formation of glass. Ding [22] finds that the pressure-mediated glasses are less energetically stable, concomitant with a denser atomic packing, and a significant increase in icosahedral short-range order. The enhanced icosahedral order is shown to be accompanied by a change in chemical short-range order. It suggests a correlation between the connectivity of the icosahedral network and the fragility in the liquid. It is debatable whether there is a clear relationship between increasing fragility and the complex string-like networks of the icosahedra. The question of whether there is an effect of pressure on complex icosahedra is still currently open. 

The high pressure processed MG has a substantially enhanced glass transition temperature, and increased density and hardness. The enhanced stability of the bulk MG originates from the change of the local atomic structure and the bonding states between atoms under high pressure. Icosahedral clusters are responsible for the increment of shear strength in MGs. In fact, icosahedra represent short-range-order which is a key feature of microstructure in MGs [22,23,24]. In simple liquids and MGs, the icosahedron is proposed to be preferred even over the faced centered cubic (FCC) and the hexagonal close-packed (HCP) clusters due to its much lower energy [24]. As a result, it needs to exert a larger stress to deform the icosahedrons than to deform other clusters [25]. If the number of icosahedrons increases in the sample, the yield stress of the material should be enhanced. Normally the string-like networks of icosahedra seem comparably too rigid to be deformed during the compression process. This means that the number and the connectivity of icosahedra are related strongly to the plastic deformation in MGs. As of today, the effects of pressure on the atomic structure and deformation behavior of glass are still unclear.

To address the aforementioned important issues, we perform extensive computer simulations to study structural and dynamical heterogeneity of binary metallic glass subjected to high pressures by calculating the number, connectivity of icosahedra, and non-Gaussian parameters (NGP). In addition, the structures and macroscopic mechanical properties of glass are also discussed in the paper by comparing stress-strain curve of glass at 0 GPa and 25 GPa.

In this paper, the molecular dynamics (MD) simulations are introduced in the first part. We present a computer simulation study of Cu_50_Zr_50_ MGs designed by pressurized quenching to provide the relationship between the structure and dynamics under pressures. By analyzing the change of the FI and their connection type, the influence of the pressure on structure is known. In the second part, the stress-strain curves are obtained by simulated loading and the difference of stress-strain curve between MGs obtained under different pressures can be explained. Previous theoretical and experimental works suggest that the low temperature relaxation of MGs could be attributed to ‘fast’ atoms which correlate with spatial heterogeneity [26,27]. In order to further study the effect of pressure on the MGs, the dynamics heterogeneity is researched, and the ‘fast’ atoms are determined in this part. Then, the relationship between the distribution of ‘fast’ atoms and shear deformation is also demonstrated. The third part is about the structural evolution of pressured MGs during compression. Since high pressure is in favor of the formation of icosahedra, which is responsible for the increment of shear strength in MGs, we try to find the types of ICOI of pressured MGs, and the changes of Voronoi polyhedrons during deformation to explore the deformation mechanism of pressured glass.

## 2. Method

**Figure 1:**
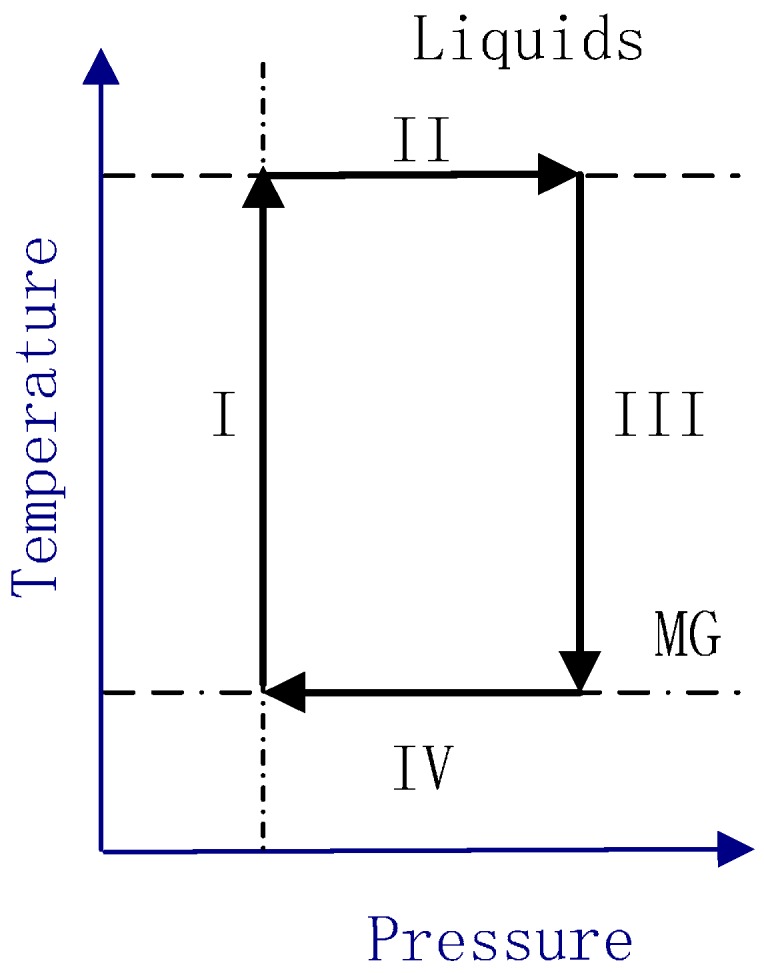
Schematic illustration of the four pathways to glass formation employed in the computer simulation studies.

We used molecular dynamics (MD) simulations of a Cu_50_Zr_50_ MG to present our study. Molecular dynamics simulations were performed by utilizing the open source code-LAMMPS [28].The embedded atom method (EAM) form ulism [29] was employed. MD simulation was performed on Cu_50_Zr_50_ MG containing 115,200 atoms, with the periodic boundary conditions (PBC) applied in all three directions. The simulation samples were obtained in four separate stages as illustrated in Figure 1: (I, II) After equilibrating at 2000 K under different external hydrostatic pressures P (P = 0.25 GPa) and temperature (NPT) ensemble for at least 2.5 ns, (III) samples were uenched to 100 K at the 10^12^ K/s, and finally (IV) the samples obtained by different pressures were equilibrated at 100 K temperature and zero pressure for 1 ns.

**Figure 2:**
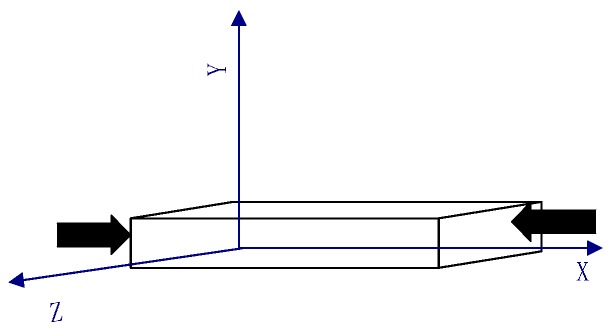
The schematic of the compression testing geometry.

After the samples quenched under different pressures were obtained, the schematic of the uniaxial compression testing is shown in Figure 2, and the PBC were imposed along the y- and z-directions. The compression loads were performed in the x-direction at a strain rate of 0.01 ns^−1^ and the maximum strains were 20% in the compressions.

## 3. Results and Discussion

### 3.1. Part A: Structural and Dynamical Heterogeneity in Liquid and Glass

**Figure 3:**
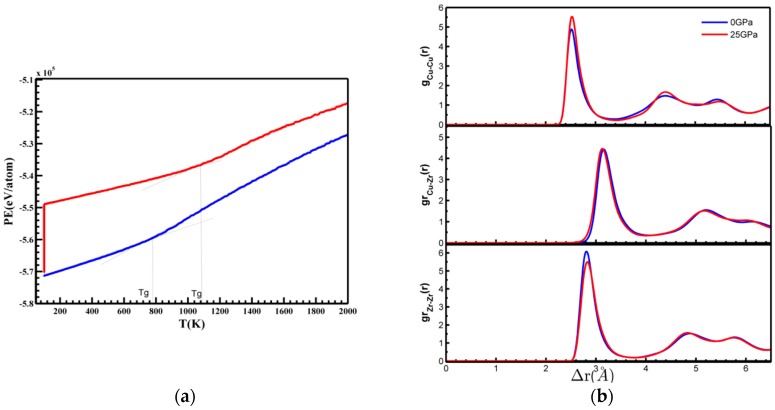
(**a**) Total energy of the two samples with applied pressures of P = 0 and 25 GPa at T = 100 K; (**b**) The pair correlation function (PCF) of the two samples with applied pressures of P = 0 and 25 GPa at T = 100 K.

The difference between the total energy as a function of temperature for various MGs obtained under different pressures at the cooling rate of 10^12^ K/s is shown in Figure 3a. The glass-transition Tg was defined as the crossover temperature between the behaviors of total energy for lower temperature and higher temperature. It is obviously in Figure 3a, the Tg of sample quenched under 25 GPa was about 1080 K, much higher than that quenched under 0 GPa, 750 K, which indicates that the high pressure was favor of the enhancement of GFA [18]. Also, the difference of the Tg showed that the atomic structure of the two samples should be different [22,30,31]. We noted that in the liquid state, the potential energy was higher for the sample at 25 GPa; however, it was quite close after glass formation and relaxation to zero pressure. Figure 3b is the total pair correlation function (PCF) of the two samples, the weaker first peak intensity of Zr–Zr, and the stronger first peak intensity of Cu–Cu at 25 GPa suggesting the decrease and increase of averaged atomic coordination numbers in the first neighbor shell.

**Figure 4:**
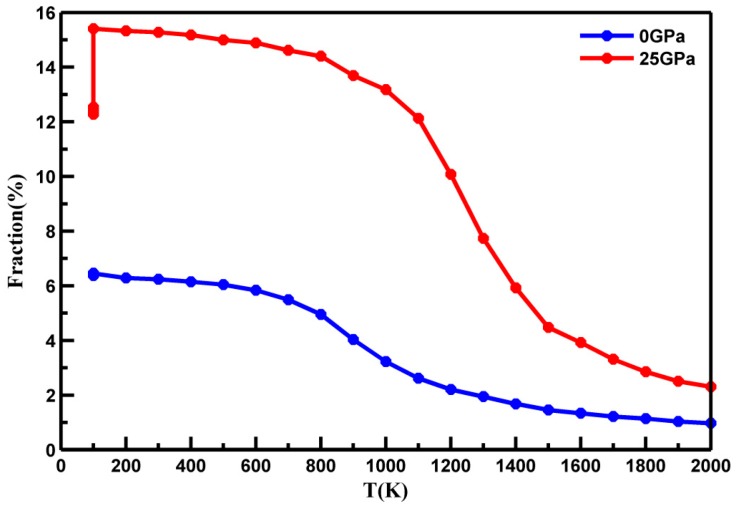
The number of full icosahedra (FI) as a function of temperature at different pressures.

To examine the differences in local structures in more detail for the metallic liquids, we studied volution of Cu-centered full icosahedra with a Voronoi index <0, 0, 12, 0> using Voronoi tessellation analysis to identify the topological packing of nearest neighbors. As in Figure 4, their quantity rose when temperature decreased, indicating that the increasing pressure enhanced the icosahedron short range order (ISRO) at the same temperature. In contrast, there was an enhanced rate of increase of ISRO with decreasing temperature during the liquid-glass transition at higher pressure than that at normal pressure. These findings indicate that, although both decreasing temperature and increasing pressure could densify the overall structure of the metallic liquid, their influences on the local structures are distinct. Pressure can slow down the dynamics of the melts, allowing glass formation to occur at higher temperatures at the same cooling rate.

Moreover, ISRO WAs found to undergo a pronounced increase during quenching close to the corresponding glass-transition temperature Tg. In Figure 3a and Figure 4, the sudden drop of the fraction of the sample quenched under 25 GPa at 100 K was caused by the pressure release of the samples at 0 GPa after being quenched. Nevertheless, the fraction of the full icosahedra <0, 0, 12, 0> of the sample quenched under 25 GPa was higher than the samples quenched under 0 GPa, which indicated that high pressure can increase the number of FI in MGs.

More importantly, Cu-Zr liquids became more fragile with higher pressure, due to the steeper rate of increase of ISRO upon undercooling, as shown in Figure 4. Recent results indicate fragile liquids are more heterogeneous, which suggests the correlation between high pressure and dynamic heterogeneity [18,32]. A popular way to quantify dynamic heterogeneity is though estimation of NGP, which can characterize the degree to which particles’ displacements deviate from the standard Gaussian distribution [33,34]. It is employed as follows:

(1) $$NGP=\frac{3\langle \Delta r^{4}(t)\rangle }{5{\langle \Delta r^{2}(t)\rangle }^{2}}-1$$

here, $\langle \Delta r^{2}(t)\rangle$ is the mean-square displacement (MSD). The advantage of MD simulations is that the trajectories of particles can always be monitored accurately. This allows us to obtain the averaged MSD $\Delta r^{2}(t)$ of an N-particle system as $\langle \Delta r^{2}(t)\rangle =\frac{1}{N}\sum_{i=1}^{N}{\left[r_{i}(t)-r_{i}(0)\right]}^{2}$; Then, the $\Delta r^{4}(t)$ is defined as $\langle \Delta r^{4}(t)\rangle =\frac{1}{N}\sum_{i=1}^{N}{\left[r_{i}(t)-r_{i}(0)\right]}^{4}$.

**Figure 5:**
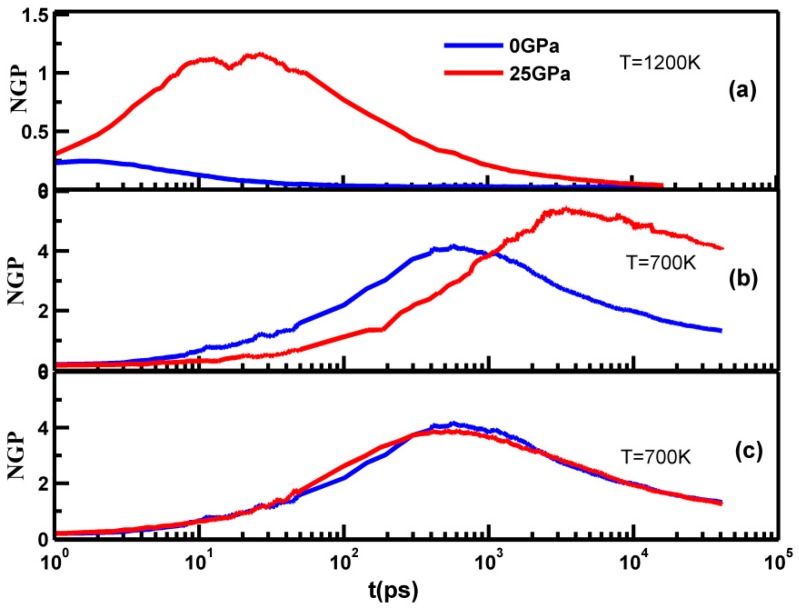
(**a**) Non-Gaussian parameter (NGP) curves of the samples quenched at 1200 K without pressure release; (**b**) NGP curves of the samples quenched at 700 K without pressure release; (**c**) NGP curves of the samples quenched at 700 K after pressure release with 0 GPa.

As shown in Figure 5, the NGP was approximately zero at the beginning relaxation period, which corresponded to the ballistic regime of MSD, due to the oscillation of particles, and then it increased sharply and decreases to zero after reaching the maximum value. The higher NGP peak of the MGs indicated more pronounced structural heterogeneity, which related to the fragility of the MGs [35,36]. It was evident that at a temperature of 1200 K, both the samples were in a liquid state, and the peak value of NGP and the corresponding time scale increased remarkably. This indicated that more heterogeneous dynamics of the sample during were being quenched under high pressure, which leads to the conclusion that the high pressure metallic liquid became more fragile. At a temperature of 700 K, shown in Figure 5b, the two sample became MGs, both peak value and corresponding time scales became larger than that at 1200 K, and this indicates that there were more heterogeneous dynamics during isobaric cooling, similar to the situation during compression. After removing the applied pressure and relaxing the samples at 700 K, the peak value at 25 GPa was a little lower than that at 0 GPa, as shown in Figure 5c, indicating that the MGs were less fragile after pressure release, quite similar to the aging treatment at a certain temperature, since the sample with the higher density at high pressure is least favored energetically. Pressure release is necessary for its further application as structural materials.

### 3.2. Part B: Dynamic Heterogeneity during Deformation

**Figure 6:**
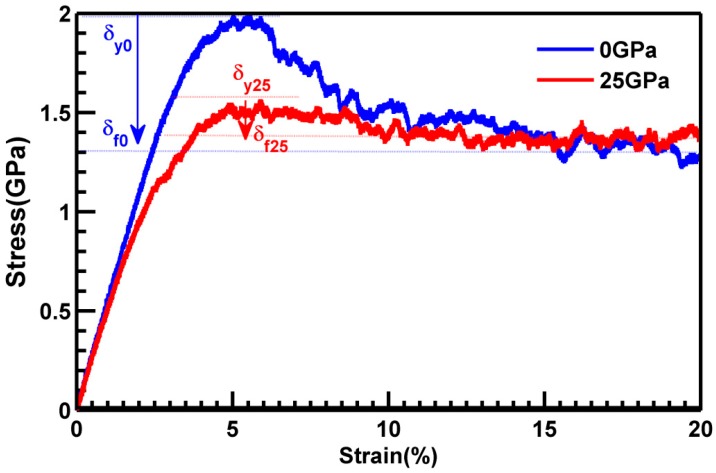
The stress-strain curve obtained from compression testing of samples quenched under 0 GPa and 25 GPa.

Since the pressured MGs was less fragile after pressure release, this encouraged us to explore the plastic deformation of the formed MGs. Figure 6 shows the uniaxial stress-strain curves of the Cu_50_Zr_50_ samples under compression. In the samples formed at 0 GPa, yielding ($\sigma_{y0})$ was observed at around 5% and was followed by a strength drop immediately after yielding. Finally, the stress saturated at the quasi-steady flow stress ($\sigma_{f0}$). The yield strength $\sigma_{y0}$ was about 2 GPa, flow stress ($\sigma_{f0}$) is about 1.3 GPa, interestingly, the pressured sample showed lower yield strength around 5%, the decrease of stress with strain was quite small, the value of flow stress ($\sigma_{f25}$) is quite similar to that of yield strength. The difference between the yield strength and the quasi-steady flow stress indicated the degree of softening during deformation, and its magnitude reflected the propensity for strain localization in the flow region. This larger strength drop in normal glass indicated that the degree of (or propensity for) strain localization was higher than that in the pressured one. The yield points decreased with increasing pressure in the MGs. The pressured samples had a lower elastic limit and were softer, which may be attributed to more ‘soft spots’ and more ‘fertile zones’ for shear transformations. Beyond yielding, the corresponding stress did not drop sharply. Previous reports demonstrated that the sharp drop of the stresses after yielding could be related to a single shear banding process [37], which in turn implied that the samples in our case could show multiple shear location states during deformation.

**Figure 7:**
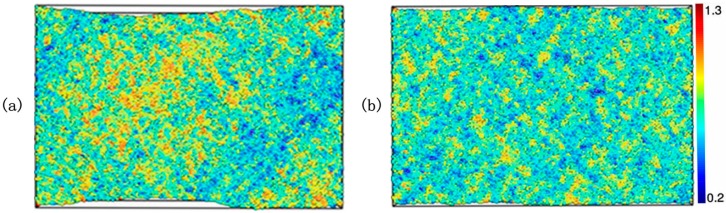
(**a**) The shear deformation of the samples quenched at 0 GPa at a strain of 20%; (**b**) The shear deformation of the samples quenched at 25 GPa at a strain of 20%.

The deformation process was monitored by the atomic local shear strains measured by $\eta_{i}^{\mathrm{Mises}}$. As a comparison, we chose samples at a strain of 20%, and took $\eta_{i}^{\mathrm{Mises}}$ as a color ruler (Figure 7a,b). Relatively large local shear strains developed not only on the free surface, but even in the early elastic regime. As the strain increased, the shear transition zones (STZ, the yellow and red parts in Figure 7a,b) propagated, coalesced, and nucleated, becoming a shear boundary. The distribution of the local shear strains was more homogeneous in pressured samples. Recent studies have demonstrated that the dynamics heterogeneity plays important roles in deformation of metallic glasses [26,36]. To further explore the plastic deformation mechanism, the atoms mobility at temperature of 700 K was calculated.

**Figure 8:**
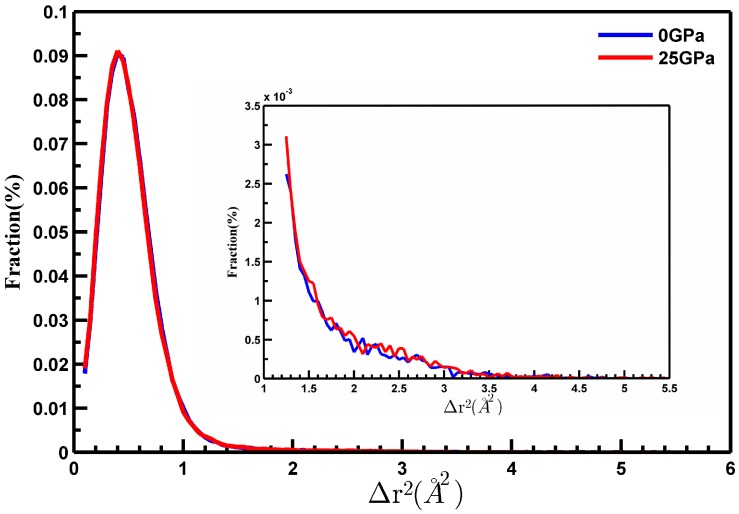
The distribution of mobility of atoms in different samples quenched at different pressures.

The atom mobility of atom *i* can be evaluated in the time interval τ* [20,21]:

(2) $$\mu_{i}(t,\tau^{*})=|r_{i}(\tau^{*}+t)-r_{i}(t)|$$

where $\mu_{i}$ represents the mobility of atom *i* and *r_i_* represents the position of atom *i*. τ* is the characteristic time scale, corresponding to the maximum of the NGP peaks. Figure 8 shows the distribution of mobility of atoms in our simulated system at temperature of 700 K. 

As shown in Figure 8, the fraction of the atomic mobility increased with increasing atomic displacement, reaching the highest value at very short displacement, and then decreasing with a long tail. The shorter displacement meant a lower mobility of atoms. The distributions of atomic mobility showed very long tails, significantly deviating from Gaussian distribution, which indicated inhomogeneous dynamics in the MGs. Obviously, the amount of atoms within a displacement of 1 Å was more pronounced compared to those with longer displacement. It was found that dynamic heterogeneity of MGs showed strong correlation with plastic deformation. The mobile atoms with larger displacement are considered as ‘fast’ atoms (FAs), while the atoms with less displacement are called ‘slow’ atoms (SAs) [18]. The FAs move cooperatively and form string-like clusters, which immobile atoms having small displacement form the cores of relatively compact clusters. The FAs region owns the liquid-like structure and bigger free volume which acts as the soft spot or flow units, where the STZs form, propagate, coalesce, and nucleate becoming shear boundary as the strain increases during the deformation of MGs. Here, the Fas were defined as the atoms whose moving distance displacement of atoms was larger than the half of first neighbor-nearest distance of 2.50 Å (the maximum of PCF g(r)) in τ time period. In the inset in the Figure 8, the number of FAs in MGs obtained at 25 GPa was 8% higher than that in MGs obtained at 0 GPa. In order to obtain more accurate arguments, we calculated the Chi-square test of the uniform distribution of the FAs in three directions (x-, y- and z-) of the box, and the result is shown in the Table 1.

The Chi-square value of the sample quenched at 25 GPa was quite a bit lower than that of the sample at 0 GPa, which indicated more homogeneous distribution of FAs at high pressure. This led to the conclusion that more uniform plastic deformation originated from the number of FAs and their distribution in the MGs. The FAs were closely related to the initiation and evolution of localized deformation unites in MGs; therefore, the research is beneficial for understanding the plastic behavior of MGs obtained at high pressure.

### 3.3. Part CStructural Evolution during Deformation

**Figure 9:**
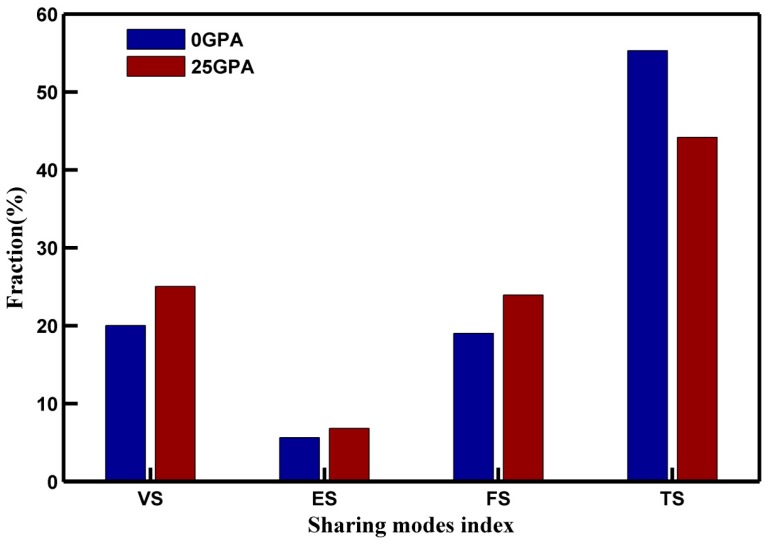
Sharing frequencies for each cluster.

To examine the deformation mechanism at pressured MGs, we analyzed the connectivity of the network formed FIs. This analysis addresses the important question of how the clusters are connected and packed before deformation. Clusters can link together to form extended clusters. These extended clusters are made of linked clusters that are interconnected to neighbor clusters by sharing their vertex (VS), edge (ES), face (FS) or volume (TS), thereby forming different types of connections [38]. The distributions of the connectivity of network formed FIs are shown in Figure 9 for MGs 0 GPa and 25 GPa respectively. 

Although the number of FIs was much larger at 25 GPa, the number of clusters with a volume connection of FIs at 25 GPa is lower than that at 0 GPa. The volume connectivity is enhanced at 0 GPa. The interpenetrating connections of icosahedra, especially the full icosahedra, produced stable string-like networks of icosahedra, which were uniquely responsible for the dynamical features of the Cu_50_Zr_50_ MGs. The full icosahedra <0, 0, 12, 0> were connected and overlapped to fill the space and formed a medium range order (MRO). The MRO clusters were characterized by an increased packing density, and high shear resistance. They became the backbone of the MGs, and were responsible for poorer plastic deformation ability.

**Figure 10:**
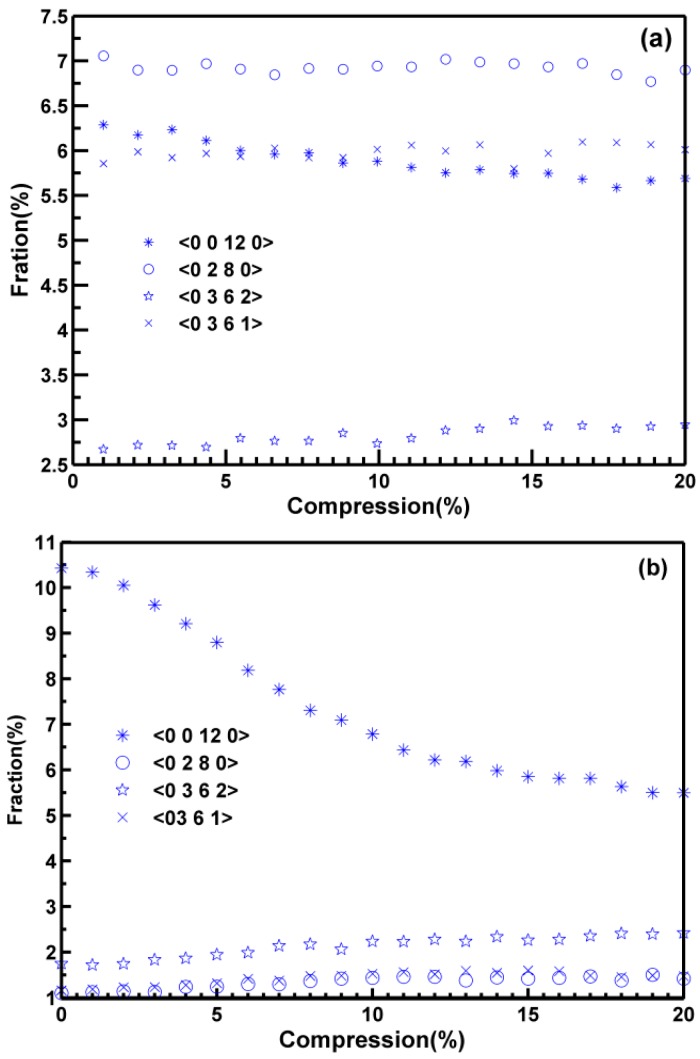
Change of the fraction of icosahedra in samples quenched under (**a**) 0 GPa (**b**) 25 GPa during compression.

We also showed the changes of main polyhedron during compressive deformation in Figure 10. The FI was represented by the Voronoi index <0, 0, 12, 0> and had 12 pentagonal faces; Triskaidecahedra hada Voronoi index <0, 3, 6, 1> and represented a polyhedron with 10 faces: six tetragonal, three pentagonal, and one hexagonal. These polyhedra have been used in previous works as an estimator of the topological changes which occur in Cu–Zr metallic glasses. For the pressured MGs, the number of <0, 0, 12, 0> polyhedra decreased sharply, as observed after 3% of strain during deformation, which was consistent with the onset of the plastic region observed in the stress-strain curve (Figure 6), and in good agreement with the fact that the FI is destroyed when plasticity begins [39]. On the contrary, the number of distorted icosahedra index <0, 2, 8, 0> and <0, 3, 6, 1> increased during compressive deformation. The polyhedron indexed <0, 2, 8, 0>could be considered as some liquid-like clusters, and the indexed <0, 3, 6, 1>could be considered as some solid-like clusters. The change of the type of icosahedra indicated that the plastic deformation in the pressured MGs involved the destruction of FIs and transformation into other clusters. Due to the lower content of complex network clusters, the backbones formed by the FIs as well as another polyhedron in the MGs were inclined to be destroyed, and the plastic deformation was improved at pressured MGs. For normal MGs, the number of <0, 0, 12, 0> polyhedral was much lower than that at 25 GPa. However, this number decreased during deformation, and it decreased rather slowly; While the number of <0, 2, 8, 0> and <0, 3, 6, 1> was almost unchanged, the number of <0, 3, 6, 2> in both samples was quite close and was constant during deformation. High pressure increased the number of the full icosahedra in the formation of MGs, while the FIs at normal MGs were more intensively connected than those in pressured MGs, which meant that the high-pressure MGs were lower anti-deforming capability and better plastic ability. All these nature are accordance with the curve in Figure 6. 

## 4. Conclusions

In conclusion, we reveal here atomic-level dynamical heterogeneity-plastic deformation relationships for MGs prepared through a pressure-mediated processing path. The isothermal compressing makes glass-forming liquids become increasingly viscous, and glass formation ability is enhanced. Meanwhile, high pressure was also found to promote more heterogeneous dynamics from NGP measurement, which is related to the more fragile state of the liquid, while the pressured MGs become less fragile after pressure release. The pressured MGs have a lower elastic limit and lower yielding strength; beyond yielding, the corresponding stress did not drop sharply, implies that the samples in our case could show multiple shear location states during deformation. Further research indicates that the distribution of the local shear strains is more homogeneous in pressured samples, which is attributed to the more uniform distribution of FAs, and which are closely related to the initiation and evolution of localized deformation units in MGs. Although the number of FI is larger, the connectivity of the icosahedrons network is weaker, the number of FI decreases sharply, and the backbone of the network is prone to destruction during deformation. Since the high pressure was in favor of the formation of glass, our findings provided some fundamentals for further investigating the structure and dynamic behaviors of metallic glass-formers related to the plastic deformation, which may shed light on understanding the mechanical properties of pressured MGs.

## Figures and Tables

**Table 1:** The results of Chi-square test of the uniform distribution of the ‘fast’ atoms in the three directions of the box.

Direction of Box	Samples Quenched at 0 GPa	Samples Quenched at 25 GPa
x-	0.0517	0.0359
y-	0.0525	0.0326
z-	0.032	0.0187
